# Anal Squamous Cell Carcinoma in African Americans with and without HIV: A Comparative Study

**DOI:** 10.24218/jcet.2015.04

**Published:** 2015-07-03

**Authors:** Carl Lokko, Jacquelyn Turner, Wonsuk Yoo, Dorian Wood, Kyra Clark, Ed Childs, Veena N. Rao, E. Shyam P. Reddy, Clarence Clark

**Affiliations:** 1 Morehouse School of Medicine, Department of Surgery, Section of Colon and Rectal Surgery, 720 Westview Drive SW, Atlanta, GA 30310-1495, USA; 2 Biostatistics Core at R-CENTER, and Department of Community Health and Preventive Medicine, Morehouse School of Medicine; 3 Morehouse School of Medicine, Department of Medicine; 4 Cancer Biology Program, Department of OB/GYN, Morehouse School of Medicine

**Keywords:** Anal carcinoma, Squamous cell carcinoma, HIV, African Americans

## Abstract

**Background:**

The incidence of anal carcinoma has increased over the last few decades especially in African Americans (AA) despite the use of highly active anti-retroviral therapy (HAART). Here, we retrospectively review oncologic outcomes of AA patients with anal squamous cell carcinoma (SCC) with and without HIV to further examine the cause of this trend.

**Materials and Methods:**

All adult AA patients diagnosed with anal SCC from 2000 to 2007 who met inclusion were examined. All patients were staged according to the American Joint Committee on Carcinoma (AJCC) sixth edition staging classification. Patients were divided into two cohorts: HIV (−) and HIV (+). Demographics, comorbidities, and oncologic outcomes were analyzed.

**Results:**

Twenty-two AA patients with anal SCC were analyzed. Fifteen (68.%) were HIV (+) and seven (32%) were negative. Seventy-four percent of HIV (+) patients were on HAART therapy at the time of diagnosis. The HIV (+) cohort was significantly younger, mostly male, and had more comorbidities compared to the negative cohort. There was no difference in tumor, nodal or metastasis (TNM) stage for both cohorts. HIV (+) patients were more likely to receive non-operative therapy. The 5-year survival rate for HIV negative and positive patients was 57% and 58%, respectively. AJCC stage was the only factor predictive of survival after performing Cox hazard proportional regression analysis, HR: 1.96 (95% CI, 0.987 to 3.881).

**Conclusions:**

In the HAART era, HIV (+) AA patients are at high risk of developing anal SCC. However, the prognosis of HIV (+) AA with anal SSC is similar to that of their HIV (−) counterparts. Carcinoma stage is the only factor predictive of survival.

## Introduction

In 2012 an estimated 6,230 people in the United States were diagnosed with anal carcinoma with 1.7 times more women diagnosed than men [1]. Squamous cell carcinoma (SCC) represents the majority of all anal carcinomas [2, 3]. Several risk factors have been associated with the development of anal carcinomas such as lifetime number of sexual partners, female gender, human papillomavirus (HPV) infection, human immunodeficiency virus (HIV) infection and men who have sex with men (MSM) [4–6]. Genetic mutation of the PIK3CA gene has also been implicated in the process of carcinogenesis of SCC [7].

Although a rare disease, the incidence of anal SCC continues to increase over the last few decades especially in African Americans [4,5,8]. In addition, African Americans, particularly men, have been found to have a lower survival rate in comparison to their Caucasian counterparts [4,8,9]. The increased prevalence of HIV infection is one factor linked to this trend with exposure to anal HPV infection being the likely inciting event for the development of SCC [4,5,9,11,12].

Conflicting studies exist regarding the effect of HIV status on the outcomes of patients with anal SCC. Moreover, few studies exist that compare oncologic outcomes of HIV positive and HIV negative African Americans with anal SCC. The primary aim of our study is to compare oncologic outcomes of HIV positive African American adult patients with anal SCC to HIV negative patients. The secondary aim is to identify other clinical variables that may contribute to survival outcomes in this cohort of patients.

## Materials and Methods

We queried a single urban hospital carcinoma registry and identified, retrospectively, all patients, ages 18 to 89, diagnosed with squamous cell anal carcinoma from 2000 to 2007. The year 2007 was selected as the last year of inclusion to ensure that all patients had at least five years of follow-up. The study was approved by the institutional review board for human subject research prior to data acquisition. All patients were staged according to the American Joint Committee on Carcinoma (AJCC) sixth edition staging classification for anal carcinoma. Patients were included only if their histologic codes were consistent with squamous-cell carcinoma of the anus and its subtypes: epidermoid, basaloid, and cloacogenic SCC. Patients were then divided into two cohorts: HIV (−) patients and HIV (+) patients. Patients with incomplete data and poor follow-up were excluded from analysis.

Overall characteristics of demographics, tumor, nodal and metastasis staging variables, treatment modality, and survival data were examined by descriptive statistics of the means and standard deviations or the medians and interquartile ranges for continuous variables and the frequencies and proportions for discrete variables. The independent t-tests or Wilcoxon rank sum tests for continuous variables and the chi-square tests or Fisher exact tests for categorical variables were applied to test the differences on oncological characteristics and distributions of treatment modalities between HIV (+) and HIV (−). Survival was calculated in months from the date of diagnosis to the date of death or last contact. Survival was estimated by the Kaplan-Meier method. The predictors of mortality were determined using a Cox regression model and/or a log-rank test. Individual predictors were considered for final analysis if the predictors’ test had a p-value of 0.25 or less. We used this elimination scheme to identify variables that could be relevant to the model while removing those that would unlikely contribute meaningfully. All statistical analysis was performed using SAS Version 9.3 (Statistical Analysis Software, Cary, NC). The level of significance was set at p < 0.05.

## Results

A total of 44 anal carcinoma patients were diagnosed at a single academic urban hospital from 2000–2007. Of these patients, 30 were identified as African American and 26 of these patients had squamous cell histology. Four of these patients had incomplete data for analysis leaving a total of 22 patients. Fifteen (68%) of the African American squamous-cell carcinoma patients were HIV positive and seven (32%) were negative (Table 1). The HIV-positive patients were on average younger (mean age of 40.5), more often male (93%), and had more comorbidities when compared to HIV-negative patients (p=0.028, Table 1). The majority of HIV-positive patients (74%) reported being on HAART therapy at the time of diagnosis. Of the comorbidities analyzed, only coronary artery disease and hemorrhoids were found to occur at a significantly higher frequency in the HIV negative group (p=0.002). Although anal herpes was diagnosed at a higher rate in HIV positive patients compared to HIV-negative patients (60% and 14%, respectively), significant difference was not obtained (p=0.074). There was no difference seen in the primary payer insurance status among the two cohorts.

In regards to oncologic outcomes, tumor staging, nodal status, and distal metastasis, among the two cohorts showed no significant difference (Table 2). HIV negative patients showed a lower rate of nodal diseases with 86.% of this cohort free of nodal metastasis compared to 60% in the HIV positive cohort, although not significantly different. Distant metastasis was seen in only one patient who was HIV positive.

Analysis of carcinoma treatment sequence and procedure variables also showed no significant difference between the two cohorts (Table 3). However, in reviewing the trends, more HIV positive patients received non-operative therapy such as concurrent chemotherapy/radiation (44%) while HIV-negative patients were more likely to undergo surgery (57%). Only a few patients had a combined therapy of surgery and chemoradiation in both HIV positive and HIV negative patients (22% versus 14%, respectively). The first treatment modality of either surgery or chemotherapy/radiation in patients receiving combined therapy showed no difference between the two cohorts. Wide local excision was the most common procedure in both groups (44% in the HIV-positive group and 57% in the HIV-negative group). Two HIV positive patients refused therapy and ultimately died of anal carcinoma.

**Figure 1:**
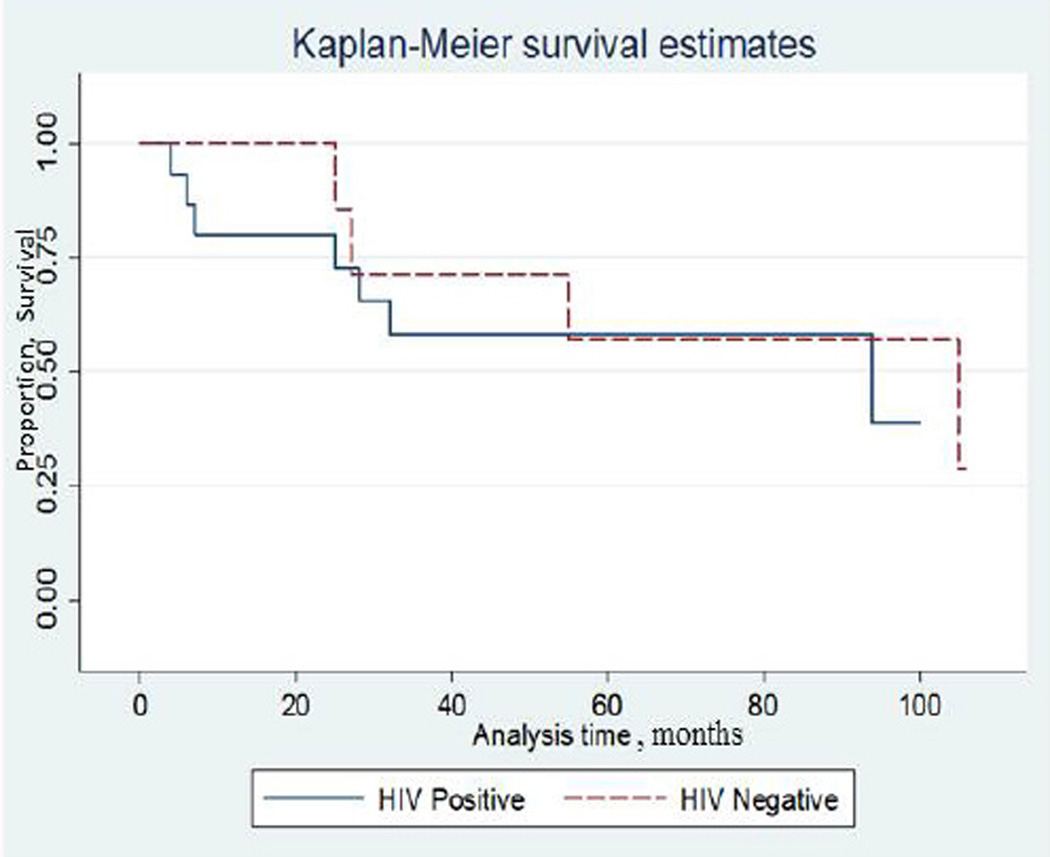
Kaplan-Meier survival estimates for HIV positive and negative patients

The combined one year and five year survival for both cohorts was 86% and 57%, respectively. At 1 year the survival was 100% and 80% for the HIV negative and positive patients, respectively. There was no significant difference in the 5-year survival for HIV negative and positive patients (57% vs. 57%, respectively) (Figure 1). Using a log rank test and univariate Cox regression analysis, sex, age, co-morbidities, AJCC staging, and treatment modality were examined. Subsequently, a Cox proportional hazard model was performed which included AJCC stage, age, and presence of anal fistula after eliminating variables that did not have a p-value less than 0.25. AJCC stage was the only factor predictive of survival after performing Cox hazard proportional regression analysis, HR: 1.96 (95% CI, 0.987 – 3.881), p=0.05.

## Discussion

HIV (+) patients are at a higher risk of developing anal SCC. Many attribute this to an increase in HPV infections and the immune compromised state of the patient suffering from HIV [11,13]. The associated risk of HIV, human papillomavirus (HPV) and anal SCC is profound enough to be an area of controversy in terms of carcinoma screening and prevention. However, in the era of Highly Active Antiretroviral Therapy, HIV positive patients are living longer and with less co-morbidities including those conditions with infectious and malignant etiologies [13]. Due to this change in the natural progression of the disease, the relationship between HIV and HIV associated comorbidities should be reevaluated. Here, the specific relationship between HIV and anal SCC is re-assessed in African American patients.

This study, unique to other studies that have evaluated HIV and anal SCC, only observed African Americans in order to obtain a more unified consortium. African Americans were specifically targeted since they tend to have a worse prognosis with anal SCC when compared to other races for reasons that are not clear [4,9,10]. There was a discrepancy in the number of patients with SCC of the anus in the HIV positive cohort (68%) compared to the number of patients in the HIV negative cohort (31%). This supports past findings that HIV positive individuals have a higher risk of developing anal SCC than HIV negative individuals [4,5,15–17].

Even though HIV positive patients are at a higher risk of developing anal SCC, we concluded that the survival rates are comparable to HIV negative patients. Specifically, the 5-year survival rates for HIV negative and HIV positive patients were 57% and 58%, respectively, which is not significantly different. Other retrospective cohort studies have demonstrated similar 5-year survival rates (61%–71%) in HIV positive patients [16,18,19,20]. In 1996, before the widespread use of HAART, poorer outcomes were demonstrated with HIV positive patients with anal SCC. For example, Stadler et al. reported a 3-year survival rate for HIV positive patients with anal SCC of 17% in the pre-HAART era [21]. Similarly, Place et al. reported a 5-year survival rate for HIV-positive patients with anal SCC of 20% in the pre-HAART era [15]. Disparities in healthcare may, in part, play a role in lack of HAART treatment received by African Americans which ultimately can affect the survival rate of HIV positive African Americans with anal SCC [22]. Further studies would be needed to asses this correlation. Some studies report poorer outcomes in AA likely due to less access to HAART therapy [22].

There were other clinical variables that did correlate with 5-year survival of SCC patients such as tumor biology, lymph node status, and the presence of metastasis. However, these variables were not significantly different between HIV positive and HIV negative patients. There was a trend towards HIV positive patients to more likely receive non-operative management (such as a combination of chemotherapy and radiation) and HIV negative patients to receive operative management. Postoperative complications such as delayed wound healing in HIV positive patients and variability in tumor size and location as well as nodal status may play a part in this treatment trend. Although not studied in this report, some studies suggest that wound healing is equivalent between the groups [23].

To our knowledge, this study is the first to specifically analyze 5-year survival rates of HIV positive and negative African American patients in the HAART era. There were several limitations with this study such as a small sample size, sampling bias, and being an observational study. Despite these limitations, our trends are comparable to trends found in previous studies. Furthermore, this observational study provides a starting point for larger studies on outcomes in HIV positive African Americans with anal SCC in the HAART era.

## Conclusion

HIV positive African Americans are at higher risk of developing anal SCC. However, survival of HIV positive African Americans is similar to HIV negative African Americans with anal SCC. With little differences seen in treatment, sequence of treatment, type of surgery, and insurance status between the two cohorts of patients, the only factor predictive of survival is stage.

## Figures and Tables

**Table 1:** Demographics of subjects by HIV Status

	HIV-Positive	HIV-Negative	p-value
	n=15	n=7	
Mean age (SD)	40.5 (±5)	54.4 (±13)	0.001
Sex (%)			0.001
Female	1 (6.7)	6 (85.7)	
Male	14 (93.3)	1 (14.3)	
Comorbidities (%)			0.028
Hepatitis (A,B,C)	11 (73.3)	3 (42.9)	0.343
Hypertension	7 (46.7)	4 (57.1)	0.376
Diabetes Mellitus	4 (26.7)	2 (28.6)	1.000
Hyperlipidemia	3 (20.0)	2 (28.6)	1.000
Coronary Artery Disease	2 (13.3)	6 (85.7)	0.002
Anal Herpes	9 (60.0)	1 (14.3)	0.074
Hemorrhoids	3 (20.0)	5 (71.4)	0.052
Anal Fistula	6 (40.0)	0 (0)	0.121
Primary Payer (%)			NS
No insurance	2 (13.3)	1 (14.3)	
Medicare	5 (33.3)	2 (28.6)	
Medicaid	7 (46.7)	3 (42.9)	
private	1 (6.7)	1 (14.3)	

* NS= not significant

**Table 2:** Oncological characteristics of subjects by HIV Status

Category	HIV-Positive	HIV-Negative	p-value
	n=15 (%)	n=7 (%)	
Primary Tumor			NS
Tis	6 (40)	3 (42.9)	
T1	1 (6.7)	1 (14.3)	
T2	1 (6.7)	1 (14.3)	
T3	6 (40)	1 (14.3)	
T4	1 (6.7)	1 (14.3)	
Nodal Status			NS
N0	9 (60)	6 (85.7)	
N1	1 (6.7)	0	
N2	3 (20)	0	
N3	2 (13.3)	1 (14.3)	
Distant Metastasis			NS
M0	14 (93.3)	7 (100)	
M1	1 (6.7)	0 (0)	

* NS= not significant

**Table 3:** Distribution of treatment modalities by HIV Status

Category	HIV-Positive	HIV-Negative	p-value
	n=15 (%)	n=7 (%)	
Treatment			NS
Surgery Only	5 (27.8)	4 (57.1)	
Chemo/XRT Only	2 (44.4)	2 (28.6)	
Surgery + Chemo/XRT	4 (22.2)	1 (14.3)	
No therapy	4 (5.6)	0 (0)	
Surgery Procedure			NS
APR	0 (0)	1 (14.3)	
WE	8 (44.4)	4 (57.1)	
APR and WE	1 (5.6)	0 (0)	
no surgery	6 (50.0)	2 (28.6)	

NS= not significant
